# Novel Designer Benzodiazepines: Comprehensive Review of Evolving Clinical and Adverse Effects

**DOI:** 10.3390/neurolint14030053

**Published:** 2022-08-22

**Authors:** Amber N. Edinoff, Catherine A. Nix, Amira S. Odisho, Caroline P. Babin, Alyssa G. Derouen, Salim C. Lutfallah, Elyse M. Cornett, Kevin S. Murnane, Adam M. Kaye, Alan D. Kaye

**Affiliations:** 1 Department of Psychiatry, Massachusetts General Hospital, Harvard School of Medicine, Boston, MA 02114, USA; 2 Department of Psychiatry and Behavioral Medicine, Louisiana State University Health Shreveport, Shreveport, LA 71103, USA; 3 Louisiana Addiction Research Center, Shreveport, LA 71103, USA; 4 School of Medicine, Louisiana State University Health Shreveport, Shreveport, LA 71103, USA; 5 School of Medicine, Louisiana State University New Orleans, New Orleans, LA 70112, USA; 6 Department of Anesthesiology, Louisiana State University Shreveport, Shreveport, LA 71103, USA; 7 Department of Pharmacology, Toxicology & Neuroscience, Louisiana State University Health Shreveport, Shreveport, LA 71103, USA; 8 Thomas J. Long School of Pharmacy and Health Sciences, Department of Pharmacy Practice, University of the Pacific, Stockton, CA 95211, USA

**Keywords:** designer drugs, benzodiazepines, flubromazolam(8-bromo-6-(2-fluorophenyl)-1-methyl-4*H*-[1,2,4]triazolo[4,3-a][1,4]benzodiazepine) clonazolam, deschloroetizolam, withdrawal, adverse effects, meclonazepam

## Abstract

As tranquilizers, benzodiazepines have a wide range of clinical uses. Recently, there has been a significant rise in the number of novel psychoactive substances, including designer benzodiazepines. Flubromazolam(8-bromo-6-(2-fluorophenyl)-1-methyl-4*H*-[1,2,4]triazolo[4,3-a][1,4]benzodiazeZpine) is a triazolo-analogue of flubromazepam. The most common effects noted by recreational users include heavy hypnosis and sedation, long-lasting amnesia, and rapid development of tolerance. Other effects included anxiolysis, muscle-relaxing effects, euphoria, loss of control, and severe withdrawals. Clonazolam, or 6-(2-chlorophenyl)-1-methyl-8-nitro-4H-[1,2,4]triazolo[4,3-α]-[1,4]-benzodiazepine, is a triazolo-analog of clonazepam. It is reported to be over twice as potent as alprazolam. Deschloroetizolam (2-Ethyl-9-methyl-4-phenyl-6H-thieno[3,2-f][1,2,4]triazolo[4,3-a][1,4]diazepine) is part of the thienodiazepine drug class, which, like benzodiazepines, stimulates GABA-A receptors. Meclonazepam ((3S)-5-(2-chlorophenyl)-3-methyl-7-nitro-1,3-dihydro-1,4-benzodiazepin-2-one) is a designer benzodiazepine with additional anti-parasitic effects. Although it has proven to be an efficacious therapy for schistosomiasis, its sedative side effects have prevented it from being marketed as a therapeutic agent. The use of DBZs has been a subject of multiple recent clinical studies, likely related to increasing presence and availability on the internet drug market and lack of regulation. Many studies have aimed to identify the prevalence of DBZs and their effects on those using them. This review discussed these designer benzodiazepines and the dangers and adverse effects that the clinician should know.

## 1. Introduction

Benzodiazepine use and abuse pose substantial burdens on the health and safety of its users in the short and long term [1]. In this regard, a review of this class of drug and their potential for harm at the population level in Canada was performed and the results indicated a need for monitoring and managing benzodiazepine overuse [1]. In recent years, there has been a significant rise in the number of novel psychoactive substances, including designer benzodiazepines (DBZs) [1]. With so many new DBZs being marketed online, the lack of research regarding their pharmacokinetics and metabolic pathways potentially makes them dangerous and unpredictable [2].

The desirable effects of benzodiazepines are achieved by modulating the central nervous system via the GABA_A_ receptor; a ligand-gated chloride-selective ion channel comprised of five subunits: two α, two β, and one γ [3]. While the endogenous neurotransmitter binds to the β subunits, benzodiazepines are positive allosteric molecules that utilize the α and γ subunits [3]. GABA induces an inhibitory effect throughout the central nervous system, including anxiolytic, hypnotic, antiseizure effects, amnestic, and muscle relaxant effects [4,5,6]. The specific physical effects are determined by the type of α subunit the drug binds [7]. Known subunits have been linked with patient-reported effects. The GABA_A_ receptors with an α1 subunit are responsible for sedative, anterograde amnesic, anti-seizure actions, and additive effects [7]. GABA_A_ receptors with an α2 subunit are responsible for anxiolytic effects, and myorelaxant actions require α2, α3, and α5 subunits [7]. These characteristic findings are useful in predicting the side effects of novel benzodiazepines.

A review of 197 trip reports of 10 different DBZs categorized and analyzed their most commonly reported effects [4]. The 10 drugs reviewed were clonazolam, deschloroetizolam, diclazepam, etizolam, flubromazepam, flubromazolam, meclonazepam, metizolam, nifoxipam, and pyrazolam [4]. Of these drugs, flubromazepam, flubromazolam, clonazolam, and meclonazepam were the most potent [4]. The least potent were deschloroetizolam, nifoxipam, metizolam, and pyrazolam [4]. Apart from nifoxipam, these rankings were confirmed by their chemical structures [4]. The most anxiolytic was reported to be pyrazolam, and the most hypnotic was flubromazolam [4]. Etizolam was the most euphoric, and flubromazolam and clonazolam were the most amnesic [4]. There were no reports of euphoria with ingestion of diclazepam and pyrazolam [4]. The synthesis of these data and disclosure of these findings to healthcare workers is vital for exploring pharmaceutical therapies and more timely diagnosis/treatment of drug toxicities. This review discussed these designer DBZs and the dangers and adverse effects that the clinician should know.

### 1.1. Benzodiazepine General Overview

With over 5% of the U.S. population filling a benzodiazepine prescription [8,9,10], setting guidelines for the safe use of this class of psychoactive drugs has become a focus of medical leaders and legislators [9]. Since their introduction over six decades ago [10], benzodiazepines have rapidly become a staple in treating several psychiatric and neurologic disorders due to their myorelaxant, anticonvulsive, anxiolytic, and hypnotic properties [11]. In certain instances, their efficacy is further improved when taken in conjunction with other widely used therapeutic drugs such as lithium, antipsychotics, and selective serotonin reuptake inhibitors (SSRIs), particularly in the setting of mania, psychotic agitation, and panic disorder, respectively [12]. Individual benzodiazepines have exhibited large differences in their half-lives [13] leading to their classification into four types: ultra-short acting, short-acting, medium acting, and long-acting based on their elimination rates [14,15,16].

A retrospective analysis of benzodiazepine prescriptions in 2008 yielded results that compared long-term prescriptions and total prescriptions by age category [8]. It was found that the percentage of benzodiazepine users increases with age, and its use was nearly twice as prevalent in women compared with men [8]. The highest prevalence of use was in 80-year-old women (11.9%) [8]. The fraction of long-term prescriptions also increased from 14.7% in the 18 to 35-year-old category to 31.4% in the 65 to 80-year-old category [8]. This presents a concern for benzodiazepine-related risks of falls, fractures, and motor vehicle collisions [17,18,19]. In the analysis of benzodiazepine use in Canada, high and/or inappropriate prescribing of these drugs was highest in older adults [1]. Rates of recreational benzodiazepine use in Canada were generally low but higher in marginalized populations with access to street drugs [1].

### 1.2. Current and Potential Clinical Use

As tranquilizers, benzodiazepines have a wide range of clinical use, most commonly for treating insomnia (temazepam) [15,20,21] and panic disorder (alprazolam, lorazepam, or clonazepam) [15,20,22,23,24,25]. Benzodiazepines are the first choice of drug for treating status epilepticus [24], a neurologic emergency that requires prompt treatment [25]. Long-acting benzodiazepines are used for treating alcohol withdrawal syndrome and associated seizures, administered immediately with a gradual reduction up to cessation [20,26,27,28,29]. Clobazam is approved for another epileptic syndrome known as Lennox–Gestaut Syndrome [28]. Clonazepam’s efficacy for obsessive-compulsive disorder is currently being investigated [30,31,32]. The potential use of alprazolam in treating post-traumatic stress disorder (PTSD) is also being explored; however, it seems that its effects are on the secondary symptom of anxiety, not the primary symptoms of the condition [33,34]. Another use of benzodiazepines in the treatment of catatonia is a neurological condition comprised of psychomotor disturbances thought to stem from a disturbance in GABA transmission [33,35].

### 1.3. Side Effects and Acute Toxicity

Prescribed benzodiazepines are considered among the safest medications, with low toxicity [13] and virtually no lethal potential if taken in isolation and as prescribed [34,36]. However, they are certainly not benign, and misuse harms the patient. Determining the appropriate benzodiazepine to prescribe requires careful consideration of the illness being treated, the patient’s age, concurrent medications, duration of treatment, and the varying potency of the drugs [15,16]. The most common side effects of benzodiazepines include dependence, rebound anxiety, memory impairment, and discontinuation syndrome [37,38]. Patients experiencing acute benzodiazepine toxicity are typically arousable with normal vital signs and classically present with central nervous system depression, slurred speech, ataxia, and altered mental status [37].

### 1.4. Dependence and Discontinuation

Despite the potential for benzodiazepine dependence, there has been a large prescription rate increase in the US over the past few decades. From 1993 to 2013, the number of US adults filling a prescription rose from 8.1 million to 13.5 million, nearly a 70% increase [7]. Although the general population is at low risk of abusing benzodiazepines, the development of dependence is worrisome. Those with a history of dependence on other sedative or hypnotic drugs are at a higher risk of developing benzodiazepine dependence; however, short-term use of benzodiazepines rarely leads to dependence [38]. Short-term dependence is typically associated with one-month use of high-potency, short half-life benzodiazepines [39,40,41]. Common withdrawal symptoms are mild and short-lasting, typically including anxiety, insomnia, restlessness, agitation, irritability, and muscle tension [42,43,44]. Such symptoms of discontinuation can be minimized by tapering the dosage instead of discontinuing suddenly [45,46,47]. However, withdrawal can also be life-threatening in some users leading to delirium and seizures. Benzodiazepine discontinuation, even gradual, in the context of long-term dependence, may lead to more severe and long-lasting symptoms [34]. Adjunctive use of certain drugs, specifically carbamazepine, buspirone, and imipramine, has increased the discontinuation success rate by reducing withdrawal symptoms [48,49,50]. However, discontinuation may not be an option for chronic benzodiazepine users who require long-term medication to continue treating their chronic anxiety or panic disorder [51,52]. When considering benzodiazepines for long-term treatment, being aware of a history of abuse is critical, especially if alternative treatments could be successful with minimal side effects and dependence.

### 1.5. Abuse and Overdose

With the increased focus on mental health literacy [51], especially with the negative psychological impacts of COVID-19 isolation [52], anxiolytic medications saw an increase in their prescription rate of 34.1% from mid-February 2019 to mid-March 2019 [53,54,55]. Problems of non-medical use of benzodiazepines mostly arise in populations who abuse other substances and possibly those who suffer from certain personality disorders [36,56,57]. In substance abuse involving benzodiazepines, opioids (54.2%) and alcohol (24.7%) [56] are the most frequent primary drug of abuse, with benzodiazepine being taken as a secondary drug to enhance the primary drug’s effects, reduce negative side effects, and alleviate discontinuation symptoms [58,59,60,61]. The increase in benzodiazepine use has a paralleled increase in benzodiazepine-associated overdose deaths, increasing from 1135 to 12,290 from 1999 to 2020 [60]. Over one-third of fatal overdoses in 2013 were associated with benzodiazepines [7], making benzodiazepines the second-most common class of prescription drugs involved in overdose deaths after opioids [62,63,64]. Individuals prescribed benzodiazepine and opiate have a 15-fold greater risk of a drug-related death than individuals not prescribed either [65,66]. This raises the question of why the proportion of people prescribed both an opioid and a benzodiazepine increased by 41% from 2002 to 2014 [65]. This phenomenon could be due to the low perceived risk associated with benzodiazepines, in line with their US classification as Schedule IV controlled substances, which usually indicates a relatively low risk for misuse [66]. Education on this matter is essential, especially for patients who are at risk of abusing either drug, since naloxone, a common treatment for opioid overdose, does not affect a benzodiazepine overdose [67,68,69]; however, flumazenil is contraindicated in patients experiencing an unknown or mixed benzodiazepine overdose [70,71]. Flumazenil is usually the reversal agent used in known benzodiazepine overdoses. What does that mean with this new designer class of benzodiazepine? These are examined in the following sections.

### 1.6. Flubromazolam

Flubromazolam (8-bromo-6-(2-fluorophenyl)-1-methyl-4*H*-[1,2,4]triazolo[4,3-a][1,4]benzodiazepine) is a triazolo-analogue of flubromazepam [70]. Despite its high potency and prolonged activity reported in recreational drug users, limited data support pharmacokinetic properties, physiologic effects, and detectability. A study of Flubromazolam‘s pharmacokinetics showed that the ingestion of 0.5 mg leads to multiple peaks in the serum concentration [71]. The onset of action is after 20–45 min with an average duration of effects of 3 to 6 h and after-effects that can last between 1 and 14 h [72]. Like other drugs of this class, flubromazolam achieves its effect by potentiating the chloride currents induced by GABA in GABA_A_ receptors [72].

An in vitro analysis of flubromazolam sought to disclose many of these parameters [73]. This analysis determined that its main metabolic pathway is hydroxylation by CYP3A4 and CYP3A5, generating α-hydroxy-flubromazolam and 4-hydroxy-flubromazolam as its predominant metabolites [73]. The drug demonstrated a high degree of protein binding and low hepatic clearance, suggesting its potential for a prolonged elimination half-life in vivo [73,74,75]. Self-reports on a Swedish online forum and case reports support the long-lasting effects of flubromazolam [76,77,78]. The most common effects noted by recreational users include heavy hypnosis and sedation, long-lasting amnesia, and rapid development of tolerance. Other effects included anxiolysis, muscle-relaxing effects, euphoria, loss of control, and severe withdrawals [76].

In one case study of an apparent flubromazolam intoxication, a 27-year-old male presented with deep coma, bilateral pinpoint unreactive pupils, acute respiratory failure, hypotension, tachycardia, and hypoxic-ischemic changes in the central nervous system [74]. Nineteen hours after ingesting 3 mg of flubromazolam, his serum and urine detected 59 ng/mL and 105 ng/mL of the drug, respectively [74]. After nine days of supportive care and around-the-clock flumazenil injections, he was stable and transferred to the Department of Neurology for a work-up of hypoxic-ischemic changes of his internal capsules bilaterally [74].

Another case study of a 36-year-old male with a long-standing history of psychiatric disorders presented to an inpatient psychiatric facility with worsening anxiety that he attempted to manage with small doses of flubromazolam [75]. After not noticing relief and shortly before presenting to the facility, he ingested 3 mg of the drug [75]. He was admitted for his sedative dependence, but the following morning, he was transferred to the emergency department and then the intensive care unit for hypotension and bradycardia (49 beats/min), appearing “nearly obtunded” [75]. Nearly 72 h after admission, his vitals normalized [75]. 

Both case reports demonstrate the prolonged activity of flubromazolam, as predicted by its high plasma protein binding [75,76,77]. One key difference in these two case reports is heart rate. Although both patients presented with hypotension, the 27-year-old male was tachycardic, while the 36-year-old male was bradycardic [76,77]. Tachycardia with concomitant hypotension is the more commonly reported side effect [76]. It is important to note that the 27-year-old reported ingesting a combination of flubromazolam and phencyclidine 48 h before hospital admission. The 36-year-old has a long list of home medications; however, his bradycardia could not be attributed to any of them [74,75].

In a self-administration study by a researcher, in vivo pharmacokinetic properties and period of detectability in biological samples were evaluated for flubromazolam [71]. The research volunteer ingested a 0.5 mg capsule of the drug and collected serum, urine, and hair samples over different time intervals. Flubromazolam was detectable in urine for up to 6.5 days, while hydroxy-flubromazolam, a metabolite, was detectable for up to 8 days [71]. As for serum levels, the first peak occurred 5 h after ingestion (7.4 ng/mL), with the greatest peak occurring post-prandially at the 8 h mark (8.6 ng/mL) [71]. A post-prandial rise could be influenced by enterohepatic cycling or delayed stomach emptying [75]. At 30 h post-ingestion, sedative effects resumed, revealing another spike in serum levels (5.2 ng/mL) [71]. The serum concentration curve generated by these findings supports the claim that flubromazolam is a long-acting benzodiazepine associated with high risks for accumulation and adverse events [71]. Immunohistochemistry failed to detect the drug in serum, but it yielded positive results for urine samples through five days post-ingestion [71]. Analysis of the volunteer’s hair revealed detection of the drug in all samples, with a peak concentration at two weeks (0.6 pg/mg) [71]. The ability to detect flubromazolam in hair after lapses in time could be vital to forensic cases.

### 1.7. Clonazolam

Clonazolam is considered a DBZ, a benzodiazepine analog found in the illicit drug markets with no medical use or FDA approval [75]. From January 2014 to December 2017, there were 234 recorded single-agent exposures to DBZs across 40 states, with the exposures increasing annually with a total increase of 330%. The second most common exposure over these four years was clonazolam, requiring hospital admission in 36% of the cases and transference to the intensive care unit in 22% of the cases [75]. Flumazenil can treat patients experiencing a clonazolam overdose [70,71] who may present with somnolence, confusion, ataxia, hyporeflexia, bradypnea, and loss of consciousness [79,80,81].

Clonazolam, or 6-(2-chlorophenyl)-1-methyl-8-nitro-4H-[1,2,4]triazolo[4,3-α]-[1,4]-benzodiazepine, is a triazolo-analog of clonazepam [80] that was first introduced in 1971 as the most active compound out of the seventeen benzodiazepines being studied in the series [81]. It has a chemical formula of C_17_H_12_ClN_5_O_2_ and an average molecular weight of 353.77 g/mol [82]. It exhibits similar properties to other benzodiazepines [83] and its users report pleasant impressions with the elimination of fear [83,84,85]. It is reportedly over twice as potent as alprazolam [2] and can be found in tablets, capsules, blotters, or liquid form [86,87].

The half-life of clonazolam is thought to be around 3.6 h and it is extensively metabolized and is mainly excreted as its amino and acetamino metabolites [86]. The main metabolites are 7-amnoclonazolam, hydroxyclonazolam, and 7-acetamindoclonazolam. All of these metabolites are eliminated in the urine. Its volume of distribution is unknown at this time. It has an onset of action between 20–60 min [86]. As with all benzodiazepines, clonazolam acts as a GABA receptor agonist, increasing the time that chloride channels are open [70]. Clonazolam may also produce loss of motor control, amnesia, respiratory depression, dizziness, and muscle relaxation [87]. The use of clonazolam has been found to antagonize the pharmacological effects of drugs like nicotine and potentiate the effects of central nervous system depressants such as alcohol [88].

### 1.8. Deschloroetizolam

Deschloroetizolam (2-Ethyl-9-methyl-4-phenyl-6H-thieno[3,2-f][1,2,4]triazolo[4,3-a][1,4]diazepine) is part of the thienodiazepine drug class, which, like benzodiazepines, stimulates GABA-A receptors [87]. Deschloroetizolam has a rapid onset of action but is thought to be half as potent as its parent compound etizolam with a direction of action that is twice as long [89]. Although it is used recreationally, very little is known regarding the characteristics of deschloroetizolam aside from what was previously stated in the last sentence. Much of what we know has been gathered from in vitro analyses, recreational user reporting, and autopsies of those who use this drug. An in vitro study was performed to determine metabolites of eight different DBZs and their close relatives, including deschloroetizolam [90]. The substances were incubated with human liver microsomes and then analyzed using mass spectrometry [90]. Deschloroetizolam yielded three different metabolites: 2 monohydroxylated metabolites and 1 dihydroxylated metabolite [90].

There are two reports contributing to our knowledge of deschloroetizolam detection in biological samples: an autopsy and a self-administration study. A forensic autopsy was performed on the corpse of a 31-year-old who used drugs and found several designer drugs, including deschloroetizolam, approximately 48 h after his death [90]. His urine and femoral blood were analyzed and revealed the presence of deschloroetizolam only in the individual’s femoral blood [90]. Although the drug was undetectable in the urine, its metabolites were easily detected in the urine [90]. The second report is from a researcher who volunteered to self-administer 6 mg of deschloroetizolam [87]. He collected oral fluid over 18 h using the NeoSal device [87]. Results showed a maximum drug concentration at 30 min with a sharp decline shortly after, but the drug was detectable in the saliva for 18 h [87]. The declining levels of the drug in the oral saliva paralleled the quick onset and decline of physical effects felt by the volunteer [87]. These physical effects included fatigue, dizziness, language disorders, and concentration trouble [87]. More information regarding the detection of deschloroetizolam is necessary to develop lab protocols for diagnosing drug toxicity cases.

Reports from recreational users and a limited number of studies reveal that deschloroetizolam is known to have a relatively fast onset, short half-life, high potency, and high bioavailability [91,92,93]. In addition to benzodiazepine effects, the physical effects of deschloroetizolam have been described as hypnotic, muscle-relaxant, amnesic, and depressant [91,92]. When combined with other sedatives and depressants, it carries a risk of increased toxicity and cross-potentiation [90].

### 1.9. Meclonazepam

Meclonazepam ((3S)-5-(2-chlorophenyl)-3-methyl-7-nitro-1,3-dihydro-1,4-benzodiazepin-2-one) is a DBZ with additional anti-parasitic effects [1,2]. Although it has proven to be an efficacious therapy for schistosomiasis, its sedative side effects have prevented it from being marketed as a therapeutic agent [93]. The only current recognized therapy for schistosomiasis is praziquantel, which comes with a risk of permanent parasite resistance [94]. A study comparing the schistosomicidal activity of clonazepam, meclonazepam, and other derivates to praziquantel demonstrated the potential efficacy of benzodiazepines as anti-parasitic agents [93]. Meclonazepam led to spastic paralysis and a 30% reduction in the worm’s body area after 1-min exposure to 10 μM [93]. To compare the lethality of praziquantel, clonazepam, and meclonazepam, they were associated with the death of all worms after five days of exposure with minimal doses of 1 μM, 20 μM, and 5 μM, respectively [93].

Although meclonazepam functions as a potent anti-parasitic agent, its sedative side effects have prevented its use. Two double-blind placebo-controlled studies reported arousal, psychomotor performance, and subjective mood following single oral doses of 1, 2, and 4 mg of meclonazepam [94,95]. The study showed a significant impairment in cognitive and psychomotor functions in all doses exceeding 1 mg [95]. Sedative effects peaked at 3 h, but moderate sedation persisted until 6 h post-ingestion [95]. New research targets involve investigating new DBZs that maintain anti-parasitic effects while mitigating the significant alterations in mood and consciousness.

While meclonazepam is a target for research, it is also recreationally used and misused [96]. A study investigating meclonazepam metabolism in both in vitro and in vivo trials identified two major metabolites suitable as biomarkers: amino-meclonazepam and acetamido-meclonazepam [96]. Recognition of biomarkers and adverse physiologic effects aid in a timely diagnosis and treatment of drug toxicities.

Pharmacokinetic data are spotty on meclonazepam. Its bioavailability is thought to be near 100% with a volume of distribution to be around 300 L and a half-life of approximately 80 h based on a single study [96]. Another single study claimed that the metabolites of meclonazepam to be amino-meclonazepam, 3-methylhydroxy-meclonazepam, and amino-3-methylhydroxy-meclonazepam; however, the authors actually provided no data that was true [96].

## 2. Clinical Studies

The use of DBZs has been a subject of multiple recent clinical studies, likely due to their increasing presence and availability on the internet drug market and lack of regulation. Many studies have aimed to identify the prevalence of DBZs and their effects on those using them. There has also been extensive research on the relation of DBZs and the impairment misuse or abuse can cause. Though little is still known, it is the hope of some of these clinical studies to better understand DBZs for many purposes, some of the greatest being identifying the prevalence of DBZ use and diagnosing and managing DBZ dependence, toxicity, and withdrawal.

### 2.1. Prevalence of Designer Benzodiazepines

Recent literature has suggested that exposure to new psychoactive substances, such as DBZ, is increasing in frequency. A US study using the National Poison Data System described the epidemiology of DBZ use. The study used data collected in the National Poison Data system between January 2014 and December 2017 to search for single-agent exposures to DBZs. The DBZs that were obtained from the system are “adinazolam, clonazolam, cloniprazepam, diclazepam, etizolam, flubromazepam, flubromazolam, meclonazepam, nifoxipam, norflurazepam, and pyrazolam” [97]. From the data, it was found that 230 single-agent exposures occurred in 40 different states during this time frame. The data also exhibited that yearly single-agent exposures increased each subsequent year, starting at 26 in 2014 and ending at 112 in 2017. Clonazolam and etizolam were the two most common culprits of single-agent exposures throughout the study. This study shows that the incidence of exposure to DBZs is rising. The jump from 26 cases in 2014 to 112 cases in 2017 is approximately a 330% increase in incidence and should prompt general awareness of DBZs and their increasing prevalence [80].

Though DBZ use is rising, users may not know they are taking DBZs. An article from 2018 published in the Journal of Applied Laboratory Medicine detailed the discovery of the DBZs clonazolam and flubromazolam in candy-like pills, specifically PEZ-like, that came from an online seller. The pills were found on an intoxicated patient when they were brought into the emergency department. The patient had a known history of drug use and was on methadone. His initial drug screen was positive for benzodiazepines, and further testing of his urine via ultra-high-performance liquid chromatography quadrupole time-of-flight mass spectrometry (UHPLC-QTof) revealed no substances concerning his clinical presentation, which was a GCS of 7 and poor oxygen saturation that required 5 L of oxygen by a mask to maintain appropriate oxygen saturation. The hospital staff then found three candy-like pills that looked like PEZ among the patient’s belongings and decided to analyze them via UHPLC-QTof. The result was two unknown peaks on the total ion chromatogram, which were further identified as clonazolam and flubromazolam based on their chemical formulas (C_17_H_13_CIN_5_O_2_ and C_17_H_13_BrFN_4_, respectively). Analyzing the PEZ-like pills provides important data that can allow faster identification of DBZ use in other patients, especially those who do not know that they used DBZs [98]. The results also provide a good learning point for clinicians in that it is important to recognize that DBZs are on the rise, and DBZ use might not even be known to the user, especially if they already abuse other drugs.

### 2.2. Difficulties in Managing and Diagnosing Designer Benzodiazepine Use

There are many challenges regarding diagnosing and managing DBZ use, especially because the clinical presentation of their misuse is not fully understood. DBZs are often not the only drugs the user is misusing at the time. It would be safe to expect that the effects of new DBZs are very similar or the same as regular benzodiazepines, such as “anxiolytic effects, hypnotic effects, sedation, muscle relaxation, and anticonvulsive effects” [80]. These suspected effects have been highlighted in earlier sections. DBZs are known to be more potent compared with traditional benzodiazepines, which can make clinical management even more difficult, especially because DBZ users often do not know exactly what drug they are taking or the dose [99,100]. A recent case report published in the Journal of Addiction Medicine investigated a 30-year-old man with opioid and sedative-hypnotic use disorder to better understand the difficulties of approaching DBZ use in patients. This patient had a history of using alcohol, prescription opioids, benzodiazepines, and intravenous heroin. The benzodiazepines he used intermittently over the years had been obtained without prescriptions and included alprazolam and clonazolam, the latter categorized as a DBZ. The patient also attended an opioid treatment program and was able to stop using IV heroin with methadone. However, he continued to use clonazolam and nonprescribed alprazolam, and his maintenance therapy with methadone was discontinued because of his continued use of benzodiazepines. This subsequently led to his return to using IV heroin, but he then self-referred himself to the clinic of the doctors who wrote this case presentation for withdrawal management. He was able to undergo successful buprenorphine induction for his heroin withdrawal management and benzodiazepine withdrawal management for three months. In the end, his cravings and worsening withdrawal symptoms resulted in a relapse of his DBZ use. He did, however, quit using heroin completely [101].

The case report mentioned in the previous paragraph highlights many challenges clinicians may face when dealing with DBZ use in their patients. One challenge is that detecting DBZs on standard drug screening used in the clinical setting may be hard. Though DBZs can be detected via liquid chromatography and mass spectrometry, it is a costly and lengthy process that is not readily available to all clinics [102]. Like the patient mentioned in the case report, users of DBZs may not be identified via routine drug screening, and clinicians may have to rely on patients to self-report DBZ use [101]. The pharmacological understanding of DBZs is also lacking. It is known that regular, prescribed benzodiazepines bind to central GABA_A_ receptors causing an inhibitory effect on neurotransmission. Though benzodiazepines bind to the same receptor, they have differing affinities for the GABA receptor. Therefore, each specific substrate (benzodiazepine) binding to the same type of receptor (GABA_A_) results in differing clinical effects [80]. DBZs, though structurally similar to regular benzodiazepines, often have high potencies and shorter to intermediate half-lives that can lead to more severe withdrawal symptoms, which in turn are more difficult to manage medically [103]. On top of more severe withdrawal symptoms, there is not enough known of the pharmacological correlation between DBZs and standard benzodiazepines to aid in withdrawal management. This means it is harder to treat DBZ use because, unlike standard benzodiazepine withdrawal management, there is no guideline for slow tapering, which has higher success rates than abrupt cessation [104].

### 2.3. Identifying Metabolites of Designer Benzodiazepines on Urine Drug Screening

Because DBZs are classified as new psychoactive substances and are not regulated like standard benzodiazepines, there have not been established ways to identify them on drug screening. Some studies have identified how these drugs are broken down and the metabolites they leave behind. One recent study in Sweden analyzed human urine samples collected from routine drug testing and from cases of intoxication to identify the main urinary metabolites of the DBZs clonazolam, meclonazepam, and nifoxipam through nano-liquid chromatography-high-resolution mass spectrometry (nano-LC-HRMS). This study relied on the STRIDA project, a project in Sweden that monitors the presence of new psychoactive substances and collects information focused on clinical symptoms and toxicity of these substances. The three previously mentioned DBZs are nitrobenzodiazepines [105]. Nitrobenzodiazepines are intermediate to long-acting and are known to be broken down by N-dealkylation to active metabolites. They may undergo another form of metabolism: nitro reduction of nitrobenzodiazepines to 7-amino compounds. The main metabolites, i.e., the metabolites found in the greatest concentration in each urine sample from the DBZs in this study, were 7-aminoclonazolam, 7-acetominomeclonazepam, and 7-acetaminonifoxipam for clonazolam, meclonazepam, and nifoxipam, respectively. These data were not unexpected, as nitrobenzodiazepines undergoing nitro reduction to 7-amino compounds are known to be a form of metabolism that they undergo. However, by identifying metabolites that are a good target for identifying specific DBZs in urine drug testing, this study could potentially set a standard for urine drug screening for DBZ abuse or misuse [105].

### 2.4. Designer Benzodiazepines and Impairment

Impairment via DBZs is also a topic of recent research. A study in Norway examined the blood concentrations of DBZs and assessed their relationships to impairment. This study used data from the analysis of blood samples collected from living individuals, which were individuals that were suspected of driving under the influence of drugs or other drug offenders, and medical/legal autopsies. These blood samples were analyzed for the presence of seven specific DBZs (clonazolam, diclazepam, etizolam, flualprazolam, flubromazepam, flubromazolam, and phenazepam) via ultra-high-performance liquid chromatography-tandem mass spectrometry (UHPLC-MS). The study was carried out over three years and three months and included 21,500 cases of driving under the influence of drugs, 5700 cases of people involved in crimes other than driving under the influence, and 6500 cases taken from autopsies. The previously listed seven DBZs were detected in 575 of all cases in the study, the majority of which involved living individuals (554 cases) [100].

Of the living cases, most users were between the ages of 20 and 44 years. Men also made up a great majority of users in the living cases, which was 87%. The prevalence of detected DBZs found in the living cases from most prevalent to least prevalent were: diclazepam (334 cases), phenazepam (138 cases), etizolam (40 cases), clonazolam (22 cases), flubromazolam (20 cases), flualprazolam (10 cases), and flubromazepam (5 cases). Of the living cases, there were 25 cases where DBZs were the only drug detected, or if other drugs were detected, their concentrations were deemed irrelevant to cause impairment. Of these 25 cases, 24 were related to driving under the influence of drugs, and in 16 of those cases, diclazepam was the detected DBZ. Concentrations of the detected DBZs in these 25 cases ranged from 0.0051 to 0.26 mg/L. For each mono-DBZ use case, a clinical test of impairment (CTI) was performed, and eight were moderately impaired, six were not impaired, six were mildly impaired, and five were considerably impaired. The study also found a significant correlation between the level of impairment and the concentration of diclazepam detected. The other drugs (etizolam, flualprazolam, flubromazepam, and phenazepam) accounted for 9 of the 25 mono cases. However, there were still too few to perform statistical analysis [100].

Among the 6500 autopsy cases, the DBZs etizolam, flubromazolam, phenazepam, and diclazepam were detected in 21 cases. Diclazepam, as also seen in the living cases, was the most prevalent DBZ detected. Overall, the concentrations of the DBZs detected were similar to the living cases, apart from flubromazolam. There was only one case of mono DBZ use found among the 21. These results did differ from the living cases in that the ages ranged from 20 to 59 years old, and almost half of the cases were women. The causes of death were not disclosed to the study’s authors because of data protection regulations [100].

The results of this study could indicate that the concentrations of DBZs commonly seen in forensic cases are associated with impairment. There are limitations to this study, most of those being due to a lack of information. The study did not account for the time of consumption of the DBZs or the doses consumed, and it also did not identify if the cases had a history of DBZ use or other drug use. Despite the limitations, the study is strong in that it is one of the first pieces of literature to dive into the relationship between DBZ use and impairment [7].

Another study in the United States assessed many cases of impaired driving that could be attributed to DBZ use, specifically etizolam and flubromazolam. The study found that in 12 cases of drivers pulled over for impaired driving in Sedgwick County, Kansas, etizolam was detected in three cases, with blood concentrations ranging from 40 to 330 ng/mL, and flubromazolam was detected in nine, with blood concentrations ranging from 7.0 to 31 ng/mL. In all cases, at least one other drug was detected, such as tetrahydrocannabinol (THC) or methamphetamine. Of the 12 cases, only 2 gave a positive immunoassay result, and the other 10 had their blood sampled. In one, a urine sample was analyzed through liquid chromatography-mass spectrometry to detect etizolam or flubromazolam, as they were above the negative control but also less than the calibrator value of the immunoassay [106]. The data in this study prove concerning, as it suggests that DBZs require a higher dose to be detected than it does to cause significant impairment. The concept that DBZ use can go undetected raises a red flag regarding public safety. Thus, this study should prompt further research into DBZs and their pharmacological effects so that regulations and standards can be set to manage, monitor, and discourage their use [106]. Table 1 summarizes the clinical findings in the literature of these different designer drugs.

## 3. Conclusions

Since the discovery of chlordiazepoxide in the 1960s, benzodiazepines have gained popularity due to their low toxicity and high safety profile compared with their predecessors, barbiturates. They are now routinely used to treat many psychiatric and neurological conditions. By the mid-1970s, they were the most frequently prescribed medication in the United States. This trend raised concerns about benzodiazepines’ potential for abuse, dependence, and overdose, despite these drugs carrying a low risk of abuse in the general population if properly administered. Short-term use of benzodiazepines has a very low risk of developing dependence, while long-term use has an increased risk, especially when using short half-life benzodiazepines.

Related to their popularity, benzodiazepines have begun being sold via Internet shops without restrictions at very low prices. These DBZs are structurally derived from medically approved benzodiazepines; however, they have never been licensed as medical drugs in the United States or Western Europe. This imposes many challenges to toxicologists and clinicians attempting to classify the symptoms and treat patients who have ingested designer benzodiazepines. By altering the structure of the benzodiazepine, the resulting triazolo-analogs, such as flubromazolam, clonazolam, deschloroetizolam, and meclonazepam, have similar effects as medically used benzodiazepines yet little is known about their specific pharmacological properties or risks related to their use. It has been shown that designer benzodiazepines can produce marked cognitive and motor impairment after acute use and the development of dependence with chronic use. With a 330% increase in designer benzodiazepine exposure from 2014 to 2017, the use of these understudied drugs is growing quickly, which are dangerous to the unaware public.

Though clinicians have a good understanding of standard benzodiazepines, there is still much to learn about DBZs. Recent literature shows that DBZs have been associated with significant impairment, have higher potencies than standard benzodiazepines, and are harder to identify via screening methods routinely used for standard benzodiazepines. Further research still must be carried out on DBZs, especially regarding pharmacology, specifically safety and efficacy. Until more is known about DBZs, it is important to monitor their prevalence and update data routinely, as they have already proven to be quite dangerous and threaten public safety.

## Figures and Tables

**Table 1:** Summary of clinical studies.

Author and Year Published	Study Title and Description	Results	Conclusions
Carpenter (2019) [80]	“Designer Benzodiazepines: A Report of Exposures Recorded in the National Poison Data System” A study done in the US reviewed the National Poison Data System for single-agent DBZ exposures between the years 2014 and 2017.	230 single-agent DBZ exposures were found across 40 states between 2014 and 2017. Incidence of single-agent DBZ exposures increased yearly, and there was a 330% increase between 2014 and 2017	DBZ use is increasing, and DBZs are becoming more prevalent. This should prompt general awareness of DBZs, especially among clinicians who may have to diagnose or manage DBZ misuse.
Pope (2018) [98]	“Novel Benzodiazepines (Clonazolam and Flubromazolam) Identified in Candy-Like Pills” A case report that analyzed 3 PEZ-like pills found on an intoxicated patient that presented to the emergency department via ultra-high-performance liquid chromatography quadrupole time-of-flight mass spectrometry (UHPLC-QTof).	Two unknown peaks on the total-ion chromatogram from the UHPLC-QT of were identified as clonazolam and flubromazolam based on their chemical formulas, C_17_H_13_CIN_5_O_2_ and C_17_H_13_BrFN_4_, respectively.	It is important to realize that DBZs are newer and do not necessarily have standard ways to be detected in drug screening. This means that sometimes, as a clinician, it may take further investigation to find a source of a patient’s intoxication. Also, there must be awareness that sometimes DBZs are abused without users even knowing they are consuming a DBZ.
Peng (2022) [101]	“Challenges of Diagnosing and Managing Designer Benzodiazepine Dependence and Withdrawal: A Case Report” Case report on a 30-year-old patient with a history of DBZ, benzodiazepine, and opioid use.	The patient struggled with abstinence from DBZ use, especially after cessation of DBZs and heroin use at the same time. Undergoing maintenance therapy for heroin use ultimately led to his complete cessation of heroin use. He, however, returned to DBZ abuse after 3 months of abstinence and battling cravings and withdrawal symptoms.	DBZs, which are more potent than regular benzodiazepines, may make managing dependence more challenging due to worsened withdrawal symptoms. Simultaneous use of other addictive drugs may also muddy managing withdrawal and dependence of DBZs.
Meyer (2016) [105]	“Identification of Main Human Urinary Metabolites of The Designer Nitrobenzodiazepines Clonazolam, Meclonazepam, and Nifoxipam by Nano-Liquid Chromatography-High-Resolution Mass Spectrometry for Drug Testing Purposes” Urine samples collected in instances of intoxication via the STRIDA project and urine samples collected via routine drug testing were analyzed via nano-LC-HRMS	The main urinary metabolites of clonazolam, meclonazepam, and nifoxipam were 7-aminoclonazolam, 7-acetaminomeclonazepam, and 7-acetaminonifoxipam, respectively.	Identifying the main urinary metabolites of the three studied DBZs sets a framework of reference for identifying DBZs on drug screening. This study highlights that the main urinary metabolites are what drug screening for DBZs should target, rather than their original compounds.
Heide (2020) [100]	“Blood Concentrations of Designer Benzodiazepines: Relation to Impairment and Findings in Forensic Cases” Blood samples obtained from individuals suspected of driving under the influence and other drug offenses and autopsy were analyzed for the presence of 7 specific DBZs via UHPLC-MS. A clinical test of impairment was also performed to examine the association between blood concentration of DBZs and impairment. This was done from 1 June 2016 to 30 September 2019.	DBZs were detected in 575 cases out of approximately 33,700 cases. Of those 575 cases, 554 cases were related to driving under the influence of drugs or other drug offenses. 25 of the 554 cases were that of mono DBZ use or use of DBZ with other drugs in which the other drug was irrelevant with respect to impairment. Of the 7 specific DBZs (clonazolam, diclazepam, etizolam, flualprazolam, flubromazepam, flubromazolam, and phenazepam) diclazepam was the most prevalent.	The results of this study suggest that certain blood concentrations of DBZs may be associated with impairment, especially in the case of driving under the influence. Though more work is needed, this study can help provide a framework for interpreting situations in which one of the 7 DBZs studied are involved.
Rohrig (2021) [106]	“Driving Impairment Cases Involving Etizolam and Flubromazolam” The study consists of 12 cases involving drivers being pulled over for impaired driving in which a DBZ was detected either via immunoassay or light chromatography-mass spectrometry.	Etizolam was detected in three of the twelve cases, and flubromazolam was detected in 9 of the 12 cases. Only 2 cases resulted in detection on immunoassay. The rest of the cases had to be determined via LC-MS, as their concentration was above the negative control but below the calibrator of the immunoassay.	Without advanced testing, DBZ use can go undetected. This is particularly concerning in cases of impairment/driving under the influence. It suggests that a concentration of DBZs small enough to not be detected on routine screening is enough to cause significant impairment and endanger public safety.

## Data Availability

All data discussed within this manuscript is available on PubMed.
